# Elucidation of the Correlation between Heme Distortion and Tertiary Structure of the Heme-Binding Pocket Using a Convolutional Neural Network

**DOI:** 10.3390/biom12091172

**Published:** 2022-08-24

**Authors:** Hiroko X. Kondo, Hiroyuki Iizuka, Gen Masumoto, Yuichi Kabaya, Yusuke Kanematsu, Yu Takano

**Affiliations:** 1 Faculty of Engineering, Kitami Institute of Technology, 165 Koen-cho, Kitami 090-8507, Japan; 2 Graduate School of Information Sciences, Hiroshima City University, 3-4-1 Ozukahigashi Asaminamiku, Hiroshima 731-3194, Japan; 3 Laboratory for Computational Molecular Design, RIKEN Center for Biosystems Dynamics Research, 6-2-3 Furuedai, Suita 565-0874, Japan; 4 Graduate School of Information Science and Technology, Hokkaido University, Kita 14, Nishi 9, Kitaku, Sapporo 060-0814, Japan; 5 Information Systems Division, RIKEN Information R&D and Strategy Headquarters, 2-1 Hirosawa, Wako 351-0198, Japan; 6 Graduate School of Advanced Science and Engineering, Hiroshima University, 1-4-1 Kagamiyama, Higashi-Hiroshima 739-8527, Japan

**Keywords:** heme distortion, pocket conformation, convolutional neural network, machine learning

## Abstract

Heme proteins serve diverse and pivotal biological functions. Therefore, clarifying the mechanisms of these diverse functions of heme is a crucial scientific topic. Distortion of heme porphyrin is one of the key factors regulating the chemical properties of heme. Here, we constructed convolutional neural network models for predicting heme distortion from the tertiary structure of the heme-binding pocket to examine their correlation. For saddling, ruffling, doming, and waving distortions, the experimental structure and predicted values were closely correlated. Furthermore, we assessed the correlation between the cavity shape and molecular structure of heme and demonstrated that hemes in protein pockets with similar structures exhibit near-identical structures, indicating the regulation of heme distortion through the protein environment. These findings indicate that the tertiary structure of the heme-binding pocket is one of the factors regulating the distortion of heme porphyrin, thereby controlling the chemical properties of heme relevant to the protein function; this implies a structure–function correlation in heme proteins.

## 1. Introduction

**Figure 1:**
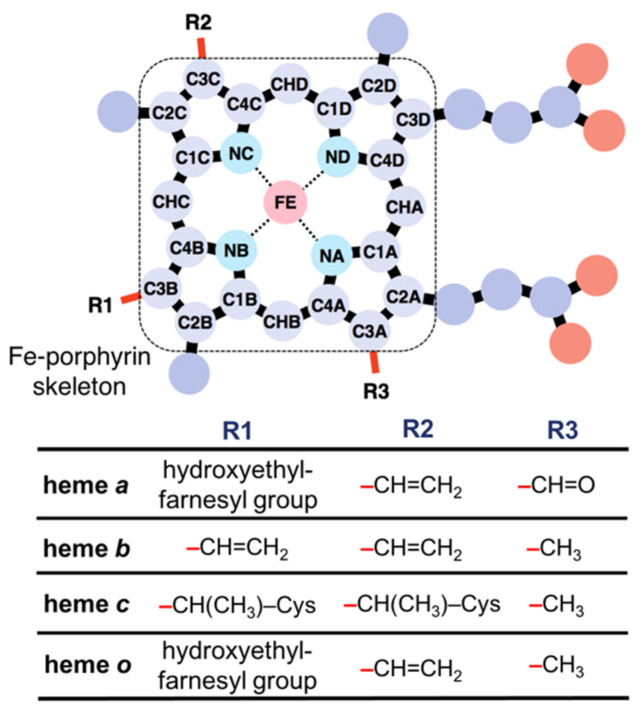
Chemical structures of hemes *a*, *b*, *c*, and *o*.

Heme proteins are a group of proteins that bind heme(s)—a complex of iron and porphyrin—to serve diverse and important biological functions. The roles of heme in heme proteins are diverse; for instance, heme acts as an electron carrier [1,2], an active site for enzymes such as oxidoreductases [3,4], an oxygen storage molecule [5,6], a ligand for proteins [7,8], and an iron storage molecule [9]. Hemophore proteins bind heme for its transport or storage [10]. Heme is classified into several types according to its peripheral groups (Figure 1), and the most common heme types are heme *b* and *c* [11,12]. Other major heme types include heme *a* and *o*, in addition to a few minor types. The key factors regulating the diverse functions of heme include the axial ligand of heme, the types of heme, the orientation of the propionate side chains, and the distortion of heme porphyrin. Since distal and proximal amino acids including axial ligands are relevant in determining heme protein functions, the role of the axial ligand and porphyrin substituents of heme has been investigated [13,14]. We have also examined the effect of the peripheral group of heme porphyrin on the redox potentials [15]. Recently, heme distortion is suggested to be correlated with the chemical properties of heme, such as redox potential and oxygen affinity [16].

Normal-coordinate structural decomposition (NSD) [17] is one of the most common methods for estimating heme porphyrin distortion. In NSD, displacement from the equilibrium structure—or distortion—is represented as a linear combination of the vibrational modes of heme porphyrin. Among these, the three lowest vibrational modes: saddling, ruffling, and doming (out-of-plane distortion), and the breathing mode (in-plane distortion) are closely correlated with its chemical properties. Bikiel et al. [18] clarified that the out-of-plane distortion tends to marginally decrease the binding affinity of heme for oxygen, while the breathing mode tends to decrease or increase it significantly. In a study on cytochrome *c*_551_, Sun et al. [19] suggested a significant role of ruffling distortion in redox control. In a systematic study, Imada et al. [20] examined the association between saddling and ruffling distortions and redox potential and indicated that saddling distortion increases the redox potential of heme, while ruffling distortion exhibits the opposite tendency. In another study, a novel distortion correlated with the chemical properties of heme was elucidated. Kanematsu et al. [21] analyzed the molecular structures of hemes in oxidoreductases and oxygen-binding proteins and successfully discovered a distortion correlated with both redox potential and oxygen affinity.

We focused on the correlation between heme distortion and protein environment, which contains proximal and distal amino acids including axial ligands, as a first step of elucidating the regulation of heme distortion caused by the environment around heme. Heme in its host protein exhibits various degrees of distortion from the isolated structure [12]. Our simulation study revealed that doming distortions in the oxygenated and deoxygenated states differ between hemoglobin and myoglobin, suggesting that the molecular structure of heme is affected by its protein environment, which controls the chemical properties of heme relevant to its protein function [22]. Some studies have reported the structural rigidity of heme-binding pockets. In addition, studies on protein structures in the apo (heme-unbound) and holo (heme-bound) states have shown that most apo–holo pairs exhibit small structural differences [23,24]. Using Brownian dynamic simulations, Sacquin-Mora et al. [25] showed that residues in the heme-binding site must be tightly anchored to realize biological functions, except for those flexible in protein function.

Our recent study using machine learning indicated a correlation between the amino acid composition of the heme-binding pocket and heme distortion along the three lowest vibrational modes [26]. Here, we investigated the correlation between the tertiary structure of the heme-binding pocket and the distortion of heme by predicting the latter from the former using a convolutional neural network (CNN). CNN is a deep learning method that has enabled breakthroughs in various computer vision tasks, such as image classification [27,28].

To this end, in the present study, we constructed a CNN model and trained it to predict heme distortion from the structure of the heme-binding pocket including the proximal and distal amino acids. We obtained high correlation coefficients for saddling, ruffling, doming, and waving(y) distortions, suggesting an association between the heme-binding pocket structure and heme distortion for these vibrational modes. Furthermore, we revealed that hemes in protein pockets with similar structures exhibit near-identical structures. These results suggest that the protein environment of the heme-binding pocket regulates the molecular structure of heme, thereby controlling the chemical properties of heme relevant to protein function. This is a first step to understand the structure–function correlation in heme proteins.

## 2. Materials and Methods

### 2.1. Data Collation on Heme Proteins and Dataset Preparation for Deep Learning

Structural information on heme proteins was extracted from the PDBx/mmCIF files downloaded from the Protein Data Bank Japan (PDBj) [29]. Briefly, we collated PDB entries, including the compound IDs (_chem_comp.id), of HEM, HEA, HEB, HEC, and HEO, and their structures at a resolution ≤ 2.0 Å via an SQL search in PDBj Mine relational database [30] (https://pdbj.org/rdb/search, accessed on 4 January 2022). Hemes with missing data in the coordinates of 25 atoms that form the Fe–porphyrin skeleton (Figure 1, upper panel) were excluded. Consequently, 6677 heme samples from 3121 unique PDB entries were selected. The Bio.PDB package [31] for BioPython version 1.78 [32] and MDTraj library version 1.9.5 [33] were used to analyze the structural data. The type of each heme molecule was determined based on peripheral groups, and the type was considered “unknown” when atoms were missing from the structural data of a heme. Protein function was classified based on structural keywords stored in the PDB entry. Details of heme protein data collection are described in our previous studies [12,26].

As a first step of elucidating the regulation of heme distortion caused by the environment around heme including axial ligands, we focused on only the protein environment for understanding the correlation between the heme-binding pocket and heme distortion. We removed the heme molecules of heme proteins in which non-amino acids are axially coordinated to heme. At this stage, 3843 samples were extracted. These samples contain proximal and distal amino acids and axially ligated amino acids, which are relevant to protein function. The axial ligands were defined as the residues or molecules with atom(s) within 3.1 Å from the Fe atom of heme. To reduce sequence redundancy in the whole dataset, we excluded protein chains with the same amino acid sequence using the PISCES server [34], yielding a nonredundant dataset. The nonredundant dataset contained 939 samples. Since even a slight difference in the amino acid sequence can affect the tertiary structure of the heme-binding pocket and distortion of heme, the threshold for sequence similarity was set to 99.99%.

The distortion of heme porphyrin was estimated using NSD [17], which is a common method for evaluating heme conformation. As mentioned earlier, NSD represents porphyrin distortion as a linear combination of distortions along the vibrational modes of heme. We calculated the equilibrium structure and vibrational modes of the Fe–porphyrin molecule using the PBE0 hybrid functional [35] with 6-31G(d) basis sets [36,37,38] and used these to estimate heme distortion. Details of the calculation are described elsewhere [12]. Only 12 vibrational modes described by Bikiel et al. [18] were considered.

### 2.2. CNN Model

**Figure 2:**
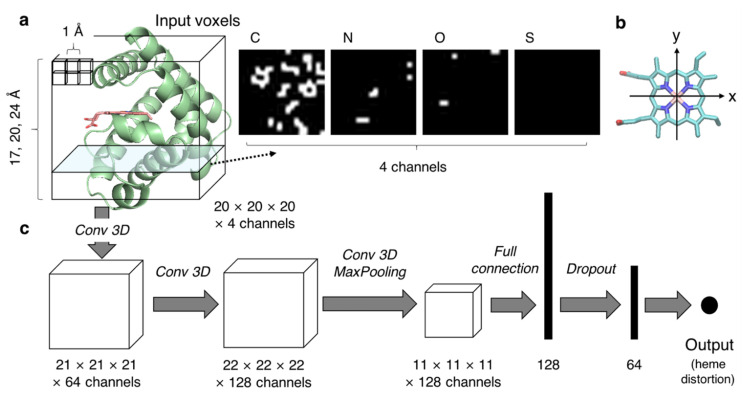
CNN model used in the present study. (**a**) A schematic diagram of input voxels. The protein backbone is represented as a green cartoon, and the heme molecule is shown as the licorice model colored in salmon. The input voxels were calculated for each atom (C, N, O, or S), as illustrated in the right panel. The heme molecule(s) were excluded in the voxel calculation. (**b**) A diagram of determination of x- and y-axes based on the coordinates of heme for the calculation of input voxels. The heme molecule is represented as the licorice model, and the atoms used for the determination of the axes are shown by dotted circles. (**c**) Layers included in the developed CNN model are shown.

Here, we constructed a CNN model whose input and output are the protein environment and the distortion of heme porphyrin, respectively, according to the following process. We converted the non-uniform protein structural data into uniform dimensional data for use as input for the CNN model. Although a set of voxels is a candidate to represent the tertiary structures of protein pockets, determining the pocket area is a problem. As shown in Figure 2a, sets of voxels in a cube-shaped inclusion region centered on the heme-binding pocket were used as input for the CNN model in the present study. The location of the cube was defined as follows. First, a least-squares plane was calculated for four atoms in the Fe–porphyrin skeleton of heme, namely CHA, CHB, CHC, and CHD (the correspondence between atom positions and names is presented in Figure 1), and defined as the xy-plane. The xy-plane was rotated such that the x-axis was parallel to the vector connecting CHA and CHC projected the least-squares plane (Figure 2b). Then, the z-axis was determined to be perpendicular to the x- and y-axes and was right-handed. The origin was determined as the mean coordinate of the four atoms: CHA, CHB, CHC, and CHD. The cube was placed such that each edge of the cube was parallel to the x-, y-, and z-axes, and the center was at the origin (0, 0, 0). The edge length was set to 17, 20, and 24 Å to examine the effect of inclusion region size on the prediction. Next, we demonstrated voxelization of the inclusion region. Using a protein structure without heme and other molecules, we generated a cubic grid with 1 Å spacing, computed whether each area was occupied by any atom, and assigned 0 (unoccupied) or 1 (occupied) to each grid. The region occupied by each atom was defined as the region within a sphere whose radius is half the length of the Van der Waals radius of the atoms—C: 1.70 Å, N: 1.55 Å, O: 1.52 Å, and S: 1.80 Å. The voxels were calculated for each atom (C, N, O, and S), and the generated data were used as an input with four channels (right panels of Figure 2a). The output was the distortion of heme porphyrin along the 12 vibrational modes (12 dimensions) or each vibrational mode (one dimension). Loss was calculated as the mean-square error between the observed (experimentally determined) and predicted values.

All CNN models were constructed and trained by using PyTorch version 1.11.0 [39]. The model used in the present study is described in Figure 2c and Table 1. The dimensions of data presented in Figure 2a,c are for the case in which the edge length in the inclusion region was 20 Å. Here, we briefly demonstrate the method commonly used in CNNs: convolution, batch normalization, activation function, pooling, and dropout. The convolutional layer, as exemplified by Conv3d in PyTorch, is the main building block of a CNN and plays a role in the extraction of local features. It selects a dot product between the values of the input voxels and filter weights. The hyperparameters of convolution include the number of output channels (number of filters), kernel size of filters, stride (number of voxels that move a filter in each step), and the presence or absence of padding (adding voxels outside the input voxels). Batch normalization, as exemplified by BatchNorm3d or BatchNorm1d in PyTorch, is a method for standardizing the inputs over mini-batches to stabilize and accelerate training by reducing the internal covariate shift. An activation function adds nonlinearity to the output and helps the neural network to learn complex patterns. Rectified linear units (ReLU), sigmoid, and hyperbolic tangent functions are common activation functions. We used the ReLU function in the present study. Pooling is a technique used to reduce feature dimensions. Max pooling, as exemplified by MaxPool3d in PyTorch, is the most commonly used pooling method. It selects the maximum value in each kernel of a feature map and generates a down-sampled feature map. The hyperparameters of max pooling include the kernel size of filters and stride. Finally, feature maps in the CNN were fully connected. Specifically, the weighted sum of outputs was computed from previous layers to obtain a specific output. A dropout layer is often added to avoid over-learning. Outputs of a randomly selected set of neurons were ignored during training. The probability of ignoring nodes is specified by a hyperparameter.

To verify the generalization performance of the model, five-fold cross-validation was performed for each vibrational mode. We did not isolate a test dataset from a cross-validation dataset because of limited data. The non-redundant dataset was split into five subsets after shuffling the samples. The following steps were performed for each subset:

1. A subset was split into validation and test datasets at a ratio of 0.2:0.8.
2. The model was trained using the remaining four subsets (training set) for 300 epochs. (In the training process, a network is trained to reduce the loss between the predicted and observed values by using an optimizer. The number of epochs indicates the number of times that training is carried out for the entire training dataset.)
3. The model with the minimum value of loss, calculated as the mean-square error, in the validation dataset was selected.
4. The resulting model was validated on the test dataset; prediction was performed by using the resulting model on the test dataset.

Adam optimizer [40] with a learning rate of 0.01 was used for training. The batch size was set to 32.

### 2.3. Clustering and Principal Component Analyses of Heme-Binding Pockets

We analyzed the three-dimensional shapes of heme-binding pockets (cavity) by using POVME 3.0 [41]. In POVME, the cavity shape of a ligand-binding protein structure can be quantified as a bit vector, each element of which indicating whether the respective grid point belongs to the ligand-binding pocket. The protein structures complexed with heme were superimposed on five atoms in heme: FE, NA, NB, NC, and ND. The coordinates of the missing atoms for proteins were generated using the AMBER LEaP program included in AmberTools version 19.0 [42]. The grid structure of the cavity was computed by using only the protein coordinates (i.e., heme and other molecules were removed). Parameters for POVME calculation were as follows: the center and radius of the inclusion sphere were set to the coordinates of the Fe atom of heme and 8.5 Å, respectively. This radius was determined according to the molecular size of heme. The distance between Fe and oxygen atoms of propionates was approximately 8.5 Å. The Tanimoto score implemented in POVME 3.0 was used to estimate the similarity between pairs of heme-binding pockets. Hierarchical clustering and principal component analysis (PCA) [43] of cavity shapes were performed by using POVME 3.0. The number of clusters was set to 35. We examined three cases of the number of clusters (15, 25, and 35) and obtained the most preferable results (many eigenvectors correlated with heme distortions) for 35.

### 2.4. Alignment of Amino Acid Sequences of Heme Proteins

We downloaded the amino acid sequences of the target heme proteins as FASTA files from PDBj (as of 4 January 2022) and extracted the sequences of 2867 protein chains in the whole dataset. Clustering was performed for the obtained sequence data using Cd-Hit [44], and threshold of sequence similarity was set to 90%.

## 3. Results and Discussion

### 3.1. Prediction of Heme Distortion from the Tertiary Structure of the Heme-Binding Pocket Using a CNN Model

We constructed a model to simultaneously predict the magnitude of distortions along the 12 vibrational modes. The edge length of the input voxel was set to 20 Å. The obtained models were assessed based on the *R*^2^ score calculated as follows:

(1) $$R^{2}=1-\frac{\sum_{i}{\left(p_{i}^{\mathrm{observed}}-p_{i}^{\mathrm{predicted}}\right)}^{2}}{\sum_{i}{\left(p_{i}^{\mathrm{observed}}-{\bar{p}}_{}^{\mathrm{observed}}\right)}^{2}},$$

where $p_{i}^{\mathrm{observed}}$ and $p_{i}^{\mathrm{predicted}}$ are the distortions of *i*th heme molecule obtained from the PDB structures and those predicted by the CNN model, respectively, and ${\bar{p}}_{}^{\mathrm{observed}}$ is the mean of heme distortion averaged over the PDB structures in the test dataset. The *R*^2^ score is a measure used to evaluate how well the model fits the regression, and its values ranges from –∞ to 1. A moderate correlation (correlation coefficient ≥ 0.6) was found between the observed and predicted values for saddling, ruffling, doming, and waving(y) distortions. Detailed prediction results are presented in Table S1, and the plot of observed and predicted values is shown in Figure S1 using results from the model with the maximum *R*^2^ score among the five cross-validation runs as an example. 

**Figure 3:**
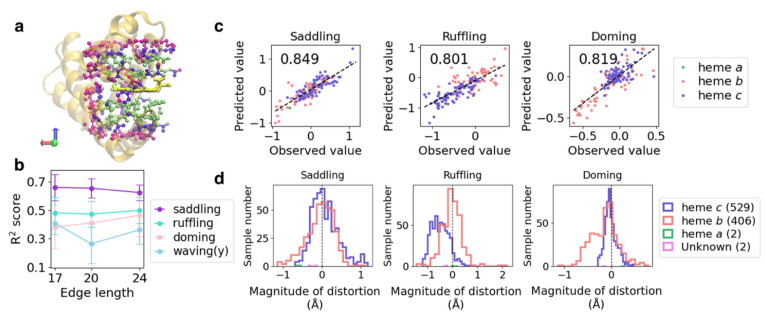
(**a**) Atoms in the inclusion region with the edge length of 17 (lime), 20.0 (violet), and 24.0 (magenta) Å, as exemplified by PDB ID: 1mba. The whole protein structure and heme molecule are shown as the orange cartoon and the yellow licorice model, respectively. (**b**) Plot of *R*^2^ scores averaged over five cross-validation runs *versus* the edge length of the input voxels for each heme distortion. (**c**) Correlation between the predicted and observed values in the test dataset of the best model among five cross-validation runs for each heme distortion. Values on the upper left of each panel represent correlation coefficients. Slate-blue, light-coral, and sea-green points indicate heme *c*, *b*, and *a*, respectively. (**d**) Distribution of saddling, ruffling, and doming distortions for each heme type in the non-redundant dataset.

To examine the effect of different edge lengths of input voxels on the prediction, we constructed models using the input voxels with edge lengths of 17, 20, and 24 Å (an example is shown in Figure 3a) for each of these four vibrational modes and calculated the corresponding *R*^2^ score. The means and standard deviations of *R*^2^ scores of the five cross-validation runs are shown in Figure 3b. Except for the waving(y) mode, changes in *R*^2^ score due to differences in the edge length of the input were very small, suggesting that information on the structure of the heme-binding pocket near the pocket surface is sufficient to predict heme distortion. In our previous study examining the correlation between the composition of amino acid residues in the heme-binding pocket and heme distortion [26], no correlation was detected for the waving(y) mode, as opposed to that for the first three vibrational modes. This might be because more detailed information on the tertiary structure of the pocket enabled us to predict even a small conformational difference.

Next, we focused on the three vibrational modes correlated with the redox potential [20] and oxygen affinity [18] of heme: the saddling, ruffling, and doming modes. The input edge length was set to 24 Å because high *R*^2^ scores were obtained for all three vibrational modes. The mean values and standard deviations of *R*^2^ scores and the root-mean-square errors (RMSEs) of the five cross-validation runs are presented in Table 2, and the corresponding correlation coefficients are listed in Table S2. Although the variation in scores among the cross-validation runs was higher for the doming distortion than for the other two distortions, we noted a strong correlation between the observed and predicted values for all three modes. In particular, high correlation coefficients were obtained for the saddling distortion, regardless of the combination of the test and training datasets; the minimum value of the correlation coefficient was 0.77. The RMSE for each magnitude of distortion averaged over five-cross validation runs showed that the prediction tended to be failed in the region with large distortion as compared with the that around 0.0 (planar structure) (Figure S2). This would be caused by the difference in the number of data; data are abundant for heme with a planar structure but few for highly distorted heme to train a CNN model. A CNN model may be improved by increasing the data of highly distorted heme.

To examine the effect of heme type on the prediction, the RMSE values for the test datasets in each cross-validation run were calculated for each heme type (Table 3). Regarding the correlation between the protein environment and heme type, only heme *c* forms covalent bonds with its host protein, causing distortion along the ruffling mode [45]. The prediction results for each heme type are shown as color-coded points in Figure 3c, and the histograms of each distortion for each heme type are presented in the lower panels of Figure 3d. Heme *c* tends to be distorted toward the ruffling mode. For ruffling and doming distortions, the RMSE values for heme *c* were almost half of those for heme *b*, suggesting a strong effect of the protein environment on heme distortion. Furthermore, we analyzed the effect of protein function on the prediction. However, the results were not sufficiently simple to observe differences in the degree of distortion for each protein function (Table S3 and Figure S3).

### 3.2. Differences in the Importance of Information Included in Subsets of Input Data

**Figure 4:**
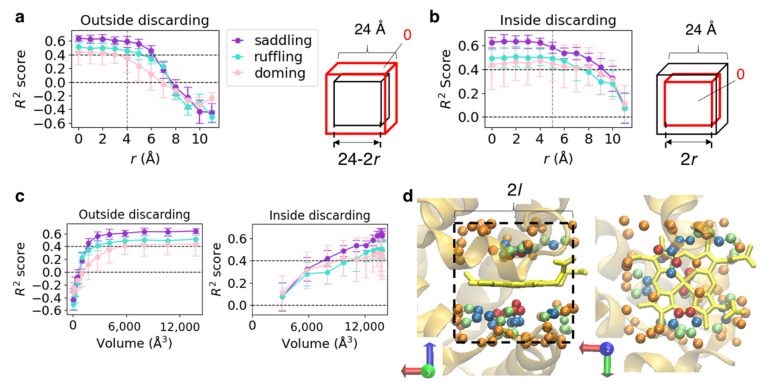
(**a**) Mean *R*^2^ scores for each vibrational mode *versus r*, which represents the distance between the faces of the red and black cubes illustrated in the right panel. The error bar shows standard deviation. The red and black cubes have an identical center, and their edges are parallel. The red cube is equivalent to the inclusion region used as the CNN input. Voxel values in the region between the red and black cubes were replaced by 0. (**b**) Mean *R*^2^ scores for each vibrational mode *versus r*. Voxel values in the red cube were replaced by 0. The coloring method is the same as that in (**a**). (**c**) *R*^2^ scores identical to those in (**a**,**b**) *versus* the volume of the region including the original information. The coloring method is the same as that in (**a**). (**d**) Atoms included in cube-shaped regions with the edge length of 2*l* are illustrated using PDB ID 1mba as an example. The dark red, lime, marine-blue, and orange spheres represent *l* = 4, 5, 6, and 7, respectively. The backbone of the host protein is represented as an orange cartoon and heme as a yellow licorice model.

To specify a region important for predicting heme distortions, we discarded the information of a specific region of input voxels and computed prediction scores using the model described in Section 3.1 (edge length of input = 24 Å). Information was discarded in two ways: “outside discarding,” which removes information from the outside (Figure 4a) and “inside discarding,” which removes information from the inside (center) (Figure 4b). First, we defined two cubes: the “outer cube” whose vertex coordinates are (±12, ±12, ±12) and the “inner cube” (right panels in Figure 4a,b). Let the coordinates of vertices of the inner cube on the “outside discarding” and “inside discarding” be (±(12 − *r*), ±(12 − *r*), ±(12 − *r*)) and (±*r*, ±*r*, ±*r*), respectively. Then, we denote the sets of voxels in the outer and inner cubes as *V*_outer_ and *V*_inner_, respectively. For “outside discarding,” the elements of *V*_outer_ − *V*_inner_ (a set of elements in *V*_outer_ but not in *V*_inner_) were replaced by 0 (0 ≤ *r* < 12, Figure 4a), that is, the information was removed from the outside of the input voxels. For the “inside discarding,” the elements of *V*_inner_ were replaced by 0 (0 ≤ *r* < 12, Figure 4b), that is, the information was removed from the inside. Since *V*_outer_ is equivalent to the input voxels used to train the CNN model, the information is intact when *r* = 0 in both cases.

Mean *R*^2^ scores obtained from predictions for each test dataset in the five cross-validation runs are shown in the left panels of Figure 4a,b. Because the change in the amount of information loss for a change in *r* was not linear and differed between “outside discarding” and “inside discarding,” we also plotted the resulting *R*^2^ scores against the volume of the region where the information remained (Figure 4c). As shown in Figure 4c, the change in *R*^2^ scores was not correlated with the amount of information but depended on the region included in the input for the prediction. With “outside discarding” (Figure 4a), the scores started decreasing significantly at *r* = 4–6 Å, where the edge length of the inner cube was 16–12 Å, reaching almost 0 at *r* = 7 Å, where the edge length of the inner cube was 10 Å. Meanwhile, for “inside discarding” (Figure 4b), the scores did not largely change at *r* = 4 Å, where the edge length of the inner cube was 8 Å, but decreased slowly at *r* = 5 Å, where the edge length of the inner cube was 10 Å. Based on these results, information from an inclusion region with the edge length of 8–16 Å is essential, while that from an inclusion region with the edge length of 8 Å is non-essential, and *A_l_* is a set of atoms included in the cubic region with edge lengths of 2*l.* Examples of *A_l_* (*l* = 4, 5, 6, and 7) are illustrated in Figure 4d using PDB ID 1mba [46]. From these results, a cubic region with the edge length (2*l*) of < 8 Å contains very few protein atoms; therefore, the structure of the pocket surface is considered to be important for the prediction.

Furthermore, we examined the impact of separation of atomic species in the input on the prediction. The CNN model shown in Figure 2c was trained and validated on a dataset with one-channel inputs (only the input dimension was different from the model in Table 1). The one-channel input was generated by calculating the logical sum (OR) of the four-channel inputs; therefore, the difference in atomic species was not considered. The results of five-fold cross-validation are presented in Table 4. The *R*^2^ score decreased in the ruffling mode, whereas no large difference was noted in the saddling and doming modes, suggesting that the steric effect was dominant for the latter two distortions.

### 3.3. Similarity of the Structure of Heme-Binding Pockets and Hemes

**Figure 5:**
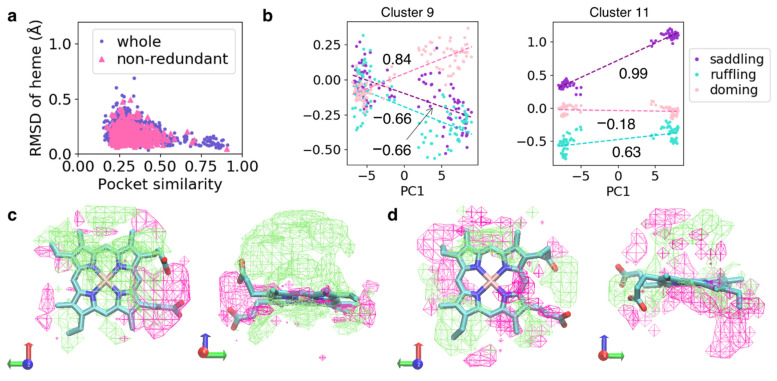
(**a**) Plot of similarity scores of cavity shapes *versus* RMSD of heme for the pairs of protein chains in the whole and non-redundant datasets. (**b**) Plot of PC1 values of cavity shapes *versus* the magnitude of distortion of heme in Clusters 9 (left panel) and 11 (right panel). Dashed lines colored in the dark-orchid, pink, and turquoise are linear regression lines for saddling, ruffling, and doming distortions, respectively. Values in the graph are correlation coefficients calculated from linear regression analysis. (**c**,**d**) First eigenvectors obtained from PCA for Clusters 9 (**c**) and 11 (**d**). Lime and magenta mesh surfaces represent the isosurfaces of +0.25 and −0.25. Structures with large PC1 values would have the cavity containing lime area but not the magenta area. Heme is represented as the licorice model. Left and right panels show the same vector viewed from different directions.

To elucidate the association between the shape of the heme-binding pocket and heme distortion, we evaluated the similarity of cavity shapes, which are a structural property of the region surrounded by the protein for pairs of protein chains. Since we considered only the structure in the vicinity of the target heme, the cavity shapes of hemes binding to a unique pocket varied in the present study. The cavity shape of the *i*th sample was represented as a bit vector using POVME, referred to as cavity vector ***v****_i_*, and the similarity score between the *i*th and *j*th samples was calculated as the Tanimoto score between ***v****_i_* and ***v****_j_*. The Tanimoto score ranges from zero to one, with one indicating identical shapes. Because the number of combinations of protein chains was very large for analysis, the pairs were randomly sampled without replacement from the whole or non-redundant dataset. The similarity score was plotted against the root-mean-square deviation (RMSD) of the heavy atoms of the heme Fe–porphyrin skeleton (Figure 5a). The pairs with high similarity scores showed small RMSD values for heme, indicating that hemes exhibit similar structures in protein pockets of similar structures. In addition, some pairs with low similarity scores showed small RMSD values for heme, indicating the lack of one-to-one correspondence between cavity shape and heme distortion.

To elucidate the simple correlation between cavity shape and heme distortion, we performed hierarchical clustering of cavity shapes for the whole dataset, followed by PCA of cavity shapes in each cluster (i.e., we conducted PCA for a group of samples with similar cavity shapes). In some clusters, we obtained eigenvectors correlated to heme distortion. Two examples with high correlation coefficients are shown in Figure 5b (Clusters 9 and 11). In Cluster 9, the PC1 values of cavity shapes were correlated with doming distortion, with a correlation coefficient of 0.84. In Cluster 11, PC1 values of cavity shapes were correlated with the saddling distortion, with a correlation coefficient of 0.99. In these examples, a difference along eigenvector led to a large difference in heme distortion, as shown in Figure 5b. The corresponding eigenvectors for Clusters 9 and 11 are shown in Figure 5c,d, respectively. In Cluster 9, the area corresponding to the element of the eigenvector with a relatively large value surrounded the Fe atom and was distributed at periphery of the heme molecule. Meanwhile, in Cluster 11, this area was distributed only at the periphery of the heme molecule. Therefore, the cavity shape of the periphery of heme may be important for saddling distortion, whereas the protein structure surrounding the Fe atom may be significant for doming distortion. Incidentally, we could not obtain features correlated with heme distortion using PCA for all samples in the whole dataset. Therefore, heme distortion is regulated by even a slight difference in cavity shape, and it is smaller than the difference in structures between all protein chains in the whole dataset (differences between clusters would be preferentially detected using PCA).

### 3.4. Similarity of the Structures of Heme-Binding Pockets between Protein Chains with Similar Amino Acid Sequences

To estimate the correlation between the amino acid sequences and cavity shapes of the pocket, we analyzed the variability of cavity shapes among homologous protein chains. By clustering protein chains in the whole dataset according to amino acid sequence, 2867 protein chains were classified into 399 clusters. From these clusters, we selected 10 clusters in the order of the number of protein chains in a cluster. Let *I* be a set of samples of cavity shapes in a cluster (the number of protein chains does not correspond to the number of heme-binding pockets because of the existence of multi-heme proteins). To estimate the dispersion of cavity shapes, we calculated the mean distance from the barycenter for cavity vector ***v****_i_* in each cluster as follows:

(2) $$N_{I}=\left|I\right|,\;\mathrm{the}\;\mathrm{number}\;\mathrm{of}\;\mathrm{samples}\;\mathrm{of}\;a\;\mathrm{set}\;I,\;$$

(3) $$\mu_{I}=\frac{1}{N_{I}}\sum_{i\in I}v_{i},$$

(4) $${\bar{d}}_{I}=\frac{1}{N_{I}}\sum_{i\in I}\|v_{i}-\mu_{I}\|,$$

where ‖·‖ represents the *L*^2^ norm.

The number of protein chains, number of samples, ${\bar{d}}_{I}$, and protein names for each cluster are presented in Table 5. Results for the whole dataset (3843 samples) are also included at the bottom of Table 5 for reference. For smaller ${\bar{d}}_{I}$ values, higher similarity was expected for cavity shapes in a cluster.

**Figure 6:**
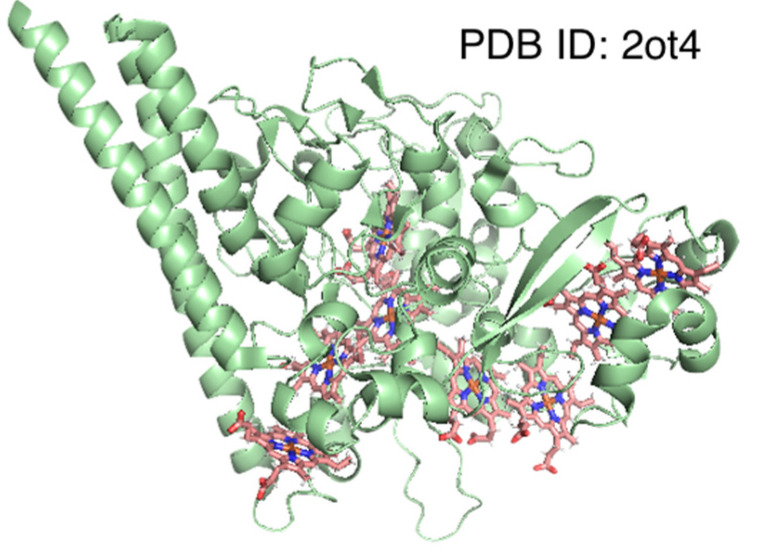
Structure of eight-heme nitrite reductase. The protein backbone is represented as a green cartoon, and hemes are shown as stick models.

For 6 of the 10 clusters, the mean distance (${\bar{d}}_{I}$) was smaller than half for the whole dataset (${\bar{d}}_{I}^{\mathrm{whole}}$), while for 2 of them (total eight clusters), the value was <60% of ${\bar{d}}_{I}^{\mathrm{whole}}$, indicating that pocket structures are similar between protein chains with near-identical amino acid sequences. The former six clusters in Table 5, whose indices are 1, 2, 3, 4, 5, and 8, include nitric oxide synthase [47], bacterioferritin [48], and hemoglobin α and β chains [49,50]. Bacterioferritin functions as an iron storage molecule or an oxidoreductase and is composed of 12 homo-dimers (i.e., 24-mer protein). Since some PDB structures only include the coordinates of the asymmetric unit, resulting in a split of heme-binding pockets [51], we excluded samples with a heme coverage of <0.6. The latter clusters (${\bar{d}}_{I}\;\leq 0.6\times {\bar{d}}_{I}^{\mathrm{whole}}$) with indices of 6 and 9 included cytochrome *c* oxidase [52] and cytochrome *c* [2], respectively. Therefore, these may be important to maintain the microstructure of the heme-binding pocket for redox control. For Cluster 7, which included dehaloperoxidases [53], ${\bar{d}}_{I}$ was slightly larger. This protein harbors a globin-like fold and functions as an oxygen storage molecule, similar to hemoglobin and peroxidase. The conformational flexibility of distal histidine increases in the deoxygenated state [54], which may explain the slightly large ${\bar{d}}_{I}$ value. Meanwhile, the ${\bar{d}}_{I}$ value of Cluster 10 was much larger than that of the other clusters. This cluster included eight-heme nitrite reductase. This enzyme possesses eight heme-binding sites, of which three are in the N-terminal domain and the remainder are in the catalytic C-terminal domain [55]. As shown in Figure 6, structural differences in these eight pockets may explain the large ${\bar{d}}_{I}$ value.

## 4. Conclusions

In the present study, we constructed a CNN model to predict heme distortion from the tertiary structure of the heme-binding pocket, which included the proximal and distal amino acids, to examine the correlation between them. The correlation between the heme-binding pocket structure and heme distortion suggests that the protein environment affects the distortion of heme and regulates its chemical properties. High *R*^2^ scores were obtained from prediction by the CNN model for saddling, ruffling, doming, and waving(y) distortions. In our previous study [26], no correlation was indicated for waving(y) distortion, as opposed to that for the remaining three distortions. This may be because detailed information on the tertiary structures of heme-binding pockets enabled us to predict even small conformational differences. These results of prediction based on partial information of the heme-binding pocket suggests that the structural information of the pocket surface is significant for the prediction of heme distortion, and the steric effect is dominant, particularly in the saddling and doming modes.

Furthermore, we examined the correlation between the shape of the cavity and molecular structure of heme and showed that hemes in protein pockets with similar structures exhibit near-identical structures. Therefore, heme distortion may be regulated by the protein environment. Finally, we estimated the correlation between the amino acid sequences and cavity shapes of heme-binding sites. The variability of cavity shapes was compared among clusters of protein chains with 90% or higher sequence similarity. We selected 10 clusters with a large number of samples and found that eight of them showed a mean distance (${\bar{d}}_{I}$) of <60% of that for the whole dataset. Therefore, pocket structures are similar among protein chains with near-identical amino acid sequences.

Overall, the tertiary structure of the heme-binding pocket is determined by the amino acid sequence of protein chain, and it is a determinant of the molecular structure of heme, thereby controlling its chemical properties, as relevant to the protein function. In this study, we considered only the protein environment including proximal and distal amino acids of heme and amino acids axially coordinated to heme. However, the ligation of non-amino acid to heme is also a determinant of the heme structure and protein function. In the future, we are going to incorporate the structural information of small molecules on heme into the CNN model. In addition, to improve the accuracy and robustness of CNN model, we attempted to increase the number of structural data by adding noise to their atomic coordinates. We showed that AlphaFold [56], a deep learning algorithm for predicting the protein tertiary structures from their amino acid sequences, can accurately predict the structure of the heme-binding pocket in heme proteins [24]. If these two challenges: (1) prediction of the location of heme-binding site from its amino acid sequence and (2) prediction of protein function from the structure of heme-binding pocket, can be overcome, the function of heme proteins may be predicted based on the amino acid sequence of the protein.

## Figures and Tables

**Table 1:** The layers and parameters of the CNN model used in this study.

Layer	Function	Filter (Kernel)	Output Dimension (Channel × Depth × Width × Height)
1	Conv3d	2 × 2 × 2 with 0-padding	64 × 21 × 21 × 21
2	Conv3d	2 × 2 × 2 with 0-padding	128 × 22 × 22 × 22
3	BatchNorm3d	-	128 × 22 × 22 × 22
4	Conv3d	2 × 2 × 2 without padding	128 × 21 × 21 × 21
5	ReLU	-	128 × 21 × 21 × 21
6	BatchNorm3d	-	128 × 21 × 21 × 21
7	MaxPool3d	2 × 2 × 2 stride: 2 × 2 × 2	128 × 10 × 10 × 10
8	Full connection	-	128,000
9	Linear	-	128
10	ReLU	-	128
11	Dropout	0.4	128
12	Linear	-	64
13	BatchNorm1d	-	64
14	ReLU	-	64
15	Linear	-	1 (or 12)

**Table 2:** The results of the prediction by the input voxels with the edge length of 24 Å. The mean value and standard deviation of *R*^2^ score, and RMSE values are listed.

	Saddling	Ruffling	Doming
*R*^2^ score (max., min.)	0.62 ± 0.05 (0.70, 0.55)	0.50 ± 0.09 (0.65, 0.39)	0.46 ± 0.15 (0.70, 0.25)
RMSE ^†^ (min., max.)	0.21 ± 0.02 (0.20, 0.24)	0.31 ± 0.04 (0.25, 0.37)	0.16 ± 0.03 (0.11, 0.20)

^†^ RMSE is shown in angstroms.

**Table 3:** The mean values and standard deviations of RMSE in angstroms between the observed and predicted values for each heme type.

Heme Type	Saddling	Ruffling	Doming
heme *c* (85.8 ± 2.7) ^†^	0.20 ± 0.01	0.22 ± 0.02	0.11 ± 0.01
heme *b* (64.2 ± 3.0)	0.22 ± 0.02	0.41 ± 0.07	0.22 ± 0.06

^†^ Values in parentheses represent the mean values of the sample numbers in the test set for five cross-validation runs.

**Table 4:** Results of prediction by the model which takes voxels with one-channel as an input.

	Saddling	Ruffling	Doming
*R*^2^ score (max., min.)	0.63 ± 0.07 (0.72, 0.53)	0.39 ± 0.10 (0.52, 0.24)	0.43 ± 0.17 (0.68, 0.17)
RMSE ^†^ (min., max.)	0.21 ± 0.02 (0.19, 0.25)	0.34 ± 0.02 (0.31, 0.37)	0.16 ± 0.03 (0.12, 0.21)

^†^ RMSE is shown in angstroms.

**Table 5:** The cluster indices, sample numbers, ${\bar{d}}_{I}$, and protein names of each cluster. The shaded rows represent the clusters with large ${\bar{d}}_{I}$.

Cluster Index	Sample Number	${\bar{d}}_{I}$	Protein Name
1	407 (407) ^†^	7.55	Nitric-oxide synthase
2	146 (146)	8.43	Hemoglobin (beta chain)
3	133 (95)	5.46	Bacterioferritin
4	103 (103)	7.72	Hemoglobin (alpha chain)
5	99 (99)	8.18	Nitric oxide synthase
6	64 (81)	8.94	Cytochrome *c* oxidase subunit 1
7	55 (55)	11.14	Dehaloperoxidase
8	50 (50)	6.41	Nitric oxide synthase oxygenase
9	47 (47)	9.84	Cytochrome *c*
10	46 (321)	14.46	Eight-heme nitrite reductase
whole dataset	3843	17.27	-

^†^ Values in parentheses represent the number of heme-binding pocket samples.

## Data Availability

The atomic coordinates of heme proteins were downloaded from PDBj (https://pdbj.org/, accessed on 4 January 2022). Our collated data on hemes are available in PyDISH: https://pydish.bio.info.hiroshima-cu.ac.jp/ (last update: 2 March 2022). For convenience, the list of PDB IDs is provided in the Supplementary Materials.

## Supplementary Materials

The following supporting information can be downloaded at https://www.mdpi.com/article/10.3390/biom12091172/s1, Table S1: Prediction results of heme distortions along the 12 vibrational modes; Figure S1: Correlation between the predicted and observed values; Table S2: Prediction results from input voxels with the edge length of 24 Å; Table S3: Mean values and standard deviations of RMSE for each protein function; Figure S2: RMSE for each magnitude of distortion; Figure S3: Distribution of saddling, ruffling, and doming distortions for each protein function.
