# Supplementary material for: Elucidation of the Correlation between Heme Distortion and Tertiary Structure of the Heme-Binding Pocket Using a Convolutional Neural Network
Source: Biomolecules. 2022 Aug 24;12(9):1172. doi: 10.3390/biom12091172 (PMC9496533; doi:10.3390/biom12091172)
Supplement: Supplementary file 1 [file biomolecules-12-01172-s001.zip › SI_kondo.pdf]

Elucidation of the correlation between heme distortion and tertiary structure of the heme-binding pocket using a convolutional neural network

Hiroko X. Kondo, Hiroyuki Iizuka, Gen Masumoto, Yuichi Kabaya, Yusuke Kanematsu, and Yu Takano

Table S1. The results of the prediction of the heme distortions along 12 vibrational modes by the input voxels with the edge length of 20 Å. The mean values and standard deviations of R^2 score, correlation coefficient, and RMSE values are listed.

	**Saddling**	**Ruffling**	**Doming**	**Waving(x)**	**Waving(y)**	**Propellering**
R^2 score	0.61 ± 0.04	0.44 ± 0.04	0.61 ± 0.05	0.05 ± 0.06	0.37 ± 0.03	0.03 ± 0.07
Correlation coef.	0.80 ± 0.02	0.68 ± 0.02	0.81 ± 0.01	0.52 ± 0.02	0.65 ± 0.02	0.34 ± 0.03
RMSE	0.22 ± 0.01	0.34 ± 0.01	0.12 ± 0.01	0.09 ± 0.00	0.10 ± 0.00	0.04 ± 0.00
	**Meso-stretching**	***N*-pyrrole stretching**	**Translation(x)**	**Translation(y)**	**Breathing**	**Rotation**
R^2 score	-0.37 ± 0.14	-0.21 ± 0.11	-0.08 ± 0.01	-0.17 ± 0.15	-0.46 ± 0.22	-0.27 ± 0.10
Correlation coef.	0.17 ± 0.02	0.21 ± 0.10	0.25 ± 0.03	0.20 ± 0.05	0.12 ± 0.07	0.02 ± 0.07
RMSE	0.09 ± 0.00	0.07 ± 0.00	0.04 ± 0.00	0.04 ± 0.00	0.13 ± 0.01	0.02 ± 0.00

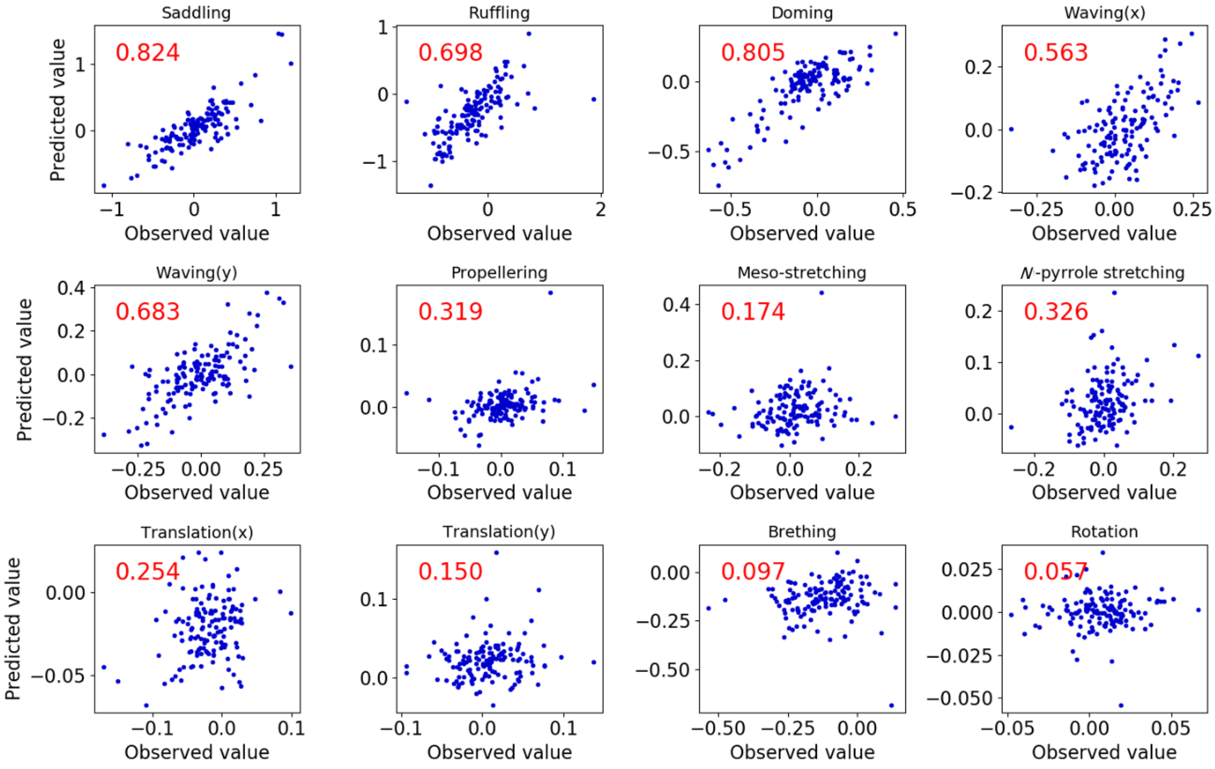

Figure S1. Correlation between the predicted and observed values in the test set of the best model among five cross-validation runs. The values colored in red in the upper left in each panel represent the correlation coefficient.

Table S2. The results of the prediction by the input voxels with the edge length of 24 Å.

	**Saddling**	**Ruffling**	**Doming**
Correlation coef.<br>(max., min.)	0.80 ± 0.03<br>(0.85, 0.77)	0.72 ± 0.06<br>(0.83, 0.63)	0.69 ± 0.11<br>(0.84, 0.52)

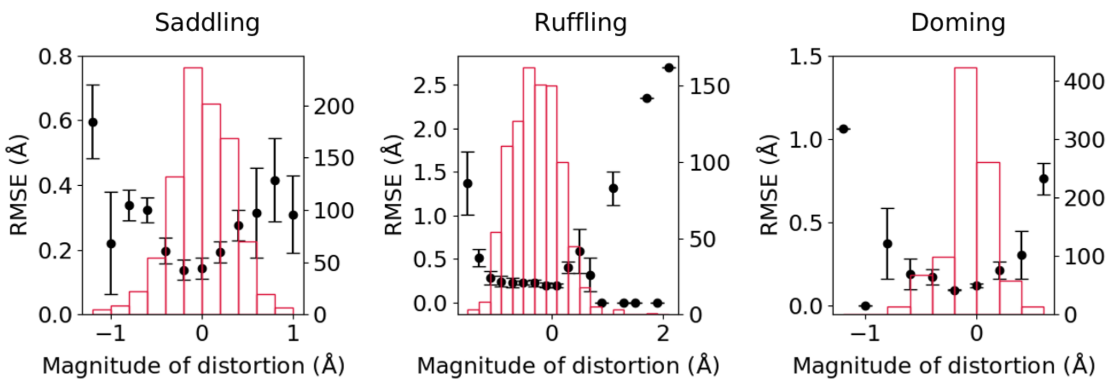

Figure S2. RMSE for each magnitude of distortion averaged over five-cross validation runs (black circle) and histogram of the number of samples in the nonredundant dataset (red bar). The error bar shows the standard deviation of RMSE for five-cross validation runs. The second y-axis (right) represents the number of samples.

Table S3. The mean values and standard deviations of RMSE between the predicted and observed values for each protein function.

Protein function	Saddling	Ruffling	Doming
Electron transfer (67.2 ± 4.9) †	0.20 ± 0.02	0.22 ± 0.02	0.10 ± 0.01
Oxidoreductase (38.6 ± 4.3)	0.25 ± 0.01	0.40 ± 0.10	0.15 ± 0.01
Oxygen binding (22.0 ± 5.2)	0.16 ± 0.03	0.25 ± 0.08	0.15 ± 0.05
Other enzymes (5.6 ± 2.3)	0.23 ± 0.09	0.47 ± 0.04	0.27 ± 0.12
Others (17.4 ± 3.4)	0.22 ± 0.02	0.36 ± 0.07	0.26 ± 0.09

† Values in parentheses represent the mean of sample numbers in the test set for five cross-validation runs.

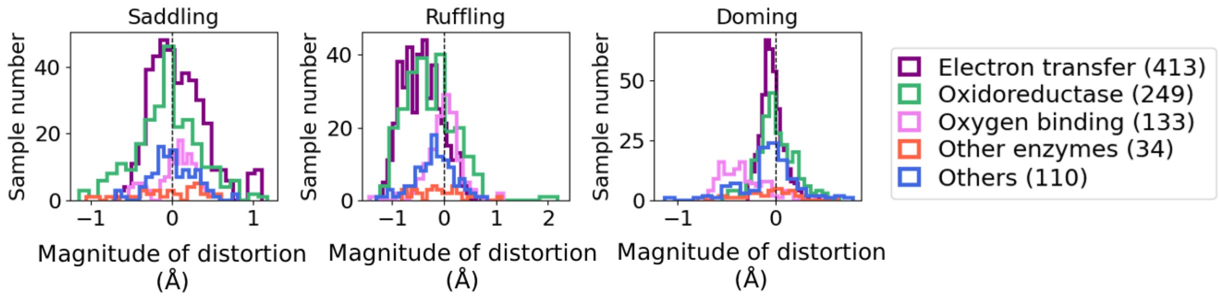

Figure S3. The magnitude of distribution of the saddling, ruffling, and doming distortions for each protein function in the non-redundant dataset.
